# Cinnamide Derivatives as Mammalian Arginase Inhibitors: Synthesis, Biological Evaluation and Molecular Docking

**DOI:** 10.3390/ijms17101656

**Published:** 2016-09-29

**Authors:** Thanh-Nhat Pham, Simon Bordage, Marc Pudlo, Céline Demougeot, Khac-Minh Thai, Corine Girard-Thernier

**Affiliations:** 1 PEPITE EA4267, University Bourgogne Franche-Comté, F-25000 Besançon, France; thanhata1@yahoo.com (T.-N.P.); simon.bordage@univ-lille2.fr (S.B.); marc.pudlo@univ-fcomte.fr (M.P.); celine.demougeot@univ-fcomte.fr (C.D.); 2 University Lille, EA 7394-ICV-Institut Charles Viollette, F-59000 Lille, France; 3 Department of Medicinal Chemistry, Faculty of Pharmacy, University of Medicine and Pharmacy at Ho Chi Minh City, 41 Dinh Tien Hoang, Dist 1, Ho Chi Minh City 700000, Vietnam; thaikhacminh@uphcm.edu.vn

**Keywords:** arginase inhibitor, cinnamide, docking, screening, structure-activity relationships

## Abstract

Arginases are enzymes that are involved in many human diseases and have been targeted for new treatments. Here a series of cinnamides was designed, synthesized and evaluated in vitro and in silico for their inhibitory activity against mammalian arginase. Using a microassay on purified liver bovine arginase (b-ARG I), (*E*)-*N*-(2-phenylethyl)-3,4-dihydroxycinnamide, also named caffeic acid phenylamide (CAPA), was shown to be slightly more active than our natural reference inhibitor, chlorogenic acid (IC_50_ = 6.9 ± 1.3 and 10.6 ± 1.6 µM, respectively) but it remained less active that the synthetic reference inhibitor *N*^ω^-hydroxy-nor-l-arginine nor-NOHA (IC_50_ = 1.7 ± 0.2 µM). Enzyme kinetic studies showed that CAPA was a competitive inhibitor of arginase with Ki = 5.5 ± 1 µM. Whereas the activity of nor-NOHA was retained (IC_50_ = 5.7 ± 0.6 µM) using a human recombinant arginase I (h-ARG I), CAPA showed poorer activity (IC_50_ = 60.3 ± 7.8 µM). However, our study revealed that the cinnamoyl moiety and catechol function were important for inhibitory activity. Docking results on h-ARG I demonstrated that the caffeoyl moiety could penetrate into the active-site pocket of the enzyme, and the catechol function might interact with the cofactor Mn^2+^ and several crucial amino acid residues involved in the hydrolysis mechanism of arginase. The results of this study suggest that 3,4-dihydroxycinnamides are worth being considered as potential mammalian arginase inhibitors, and could be useful for further research on the development of new arginase inhibitors.

## 1. Introduction

l-Arginine (l-Arg) is an amino acid involved in distinct metabolic routes for the synthesis of many different compounds including proteins, urea, polyamines, proline or nitric oxide (NO), and is consequently the substrate of various enzymes. Therefore, many studies focused on the search for bioactive compounds that are able to regulate l-arginine metabolism [1]. Nitric oxide synthase (NOS) hydrolyses l-Arg to produce l-citrulline and NO, a crucial vasorelaxant factor. Arginase (amidinohydrolase, EC 3.5.3.1), by hydrolysing l-arginine into l-ornithine and urea, plays an important role in the ammonia detoxification in mammals [2]. Interestingly, it has been shown these last few years that this enzyme also plays a crucial role in the bioavailability of l-arginine for nitric oxide synthase (NOS) by substrate competition [3]. Therefore, an increased activity of arginase is associated with various diseases by reducing the supply of l-arginine needed by NOS to produce NO, and by raising production of l-ornithine resulting in vascular structural problems [4]. In fact, l-ornithine is converted into either polyamines (putrescine, spermidine and spermine) or into proline, and these downstream products can promote cell proliferation and collagen production [4,5]. Over the last few decades, interest has grown concerning the use of arginase for therapeutic uses. Indeed, the use of arginase inhibitors has proven to be beneficial in various pathophysiological states such as hypertension [6], erectile dysfunction [7], pulmonary hypertension [8], atherosclerosis [9], diabetic renal injury [10], asthma and allergic rhinitis [11], myocardial ischemia-reperfusion injury [12], wound healing [13], and cancer [14]. The first generation of arginase inhibitors comprised analogs of *N*^ω^-hydroxy-l-arginine (NOHA), the intermediate in the production of NO from l-arginine by NOS [15,16]. These inhibitors are characterized by *N*-hydroxy-guanidium side chains. Among them, *N*^ω^-hydroxy-nor-l-arginine (nor-NOHA) is the most potent arginase inhibitor known to date [17]. Boronic acid analogs of l-arginine formed the second generation of arginase inhibitors, among which *S*-(2-boronoethyl)-l-cysteine (BEC) and 2-(*S*)-amino-6-boronohexanoic acid (ABH) are the main representative compounds [18,19]. Some of these synthetic arginase inhibitors, such as nor-NOHA, BEC and ABH, are currently available commercially. However, the use of such molecules for therapeutic purposes has several limitations: poor bioavailability and potential toxicity (BEC, and ABH) [20], very short half-life (nor-NOHA) [21], and also high cost [22]. Considering that the development of arginase inhibitors is of great therapeutic relevance in various human diseases, investigations have been undertaken in order to develop prodrugs of NOHA [23] or optimized ABH by substituting the Cα-amino acid function [12,24,25,26] and by replacing the boronic acid function [27,28,29,30,31]. Plants have been shown to be a source of promising arginase inhibitors [32]. Nevertheless, hemisynthesis has never been used for the design of new arginase inhibitors [32]. Among the natural compounds tested as potential arginase inhibitors, we previously reported that chlorogenic acid (CGA) displayed an interesting activity on bovine liver arginase I (b-ARG I) [33,34]. We therefore considered it as a potential lead compound. CGA is an ester of caffeic and quinic acids. We showed that the caffeoyl (i.e., dihydroxy cinnamoyl) part is probably more involved in the arginase inhibition than the quinoyl moiety [33,34]. Therefore, we replaced this latter moiety, containing four asymmetric carbons, in order to improve the inhibitory activity and to simplify the chemical structure. Considering that the stability of a compound is improved when its ester bond is replaced by an amide bond [35], and that the amidification with a range of phenethylamines has already been noted for their inhibitory activity on tyrosinase (which is another binuclear metalloenzyme) [36], we decided to evaluate this kind of amides for their arginase inhibition. Finally, given that several natural polyphenolic products have significant effects on arginase [34], the functionalization has been mainly focused on methoxy and hydroxyl groups which are common in such compounds. The optimal size of compounds and the contribution of each part have been explored to identify portions of the molecule that are essential for the expected biological activity.

**Figure 1:**
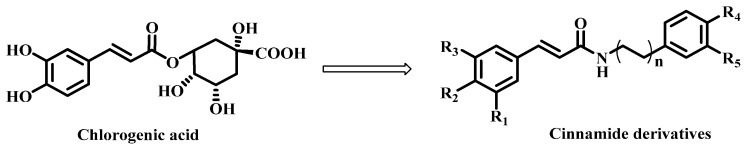
Design of cinnamide derivatives from chlorogenic acid.

The present work first aimed at synthesizing new or already known cinnamide derivatives in order to test their inhibitory property on a purified bovine liver arginase (b-ARG I) (Figure 1). Then, the most active compound of this series was evaluated on a recombinant human arginase I (h-ARG I). Additionally, enzyme-kinetic and molecular docking studies were also performed on the most active compounds and compared to CGA in order to analyze their inhibition mechanism and the protein-ligand interactions, respectively.

## 2. Results and Discussion

### 2.1. Chemistry

**Scheme 1:**
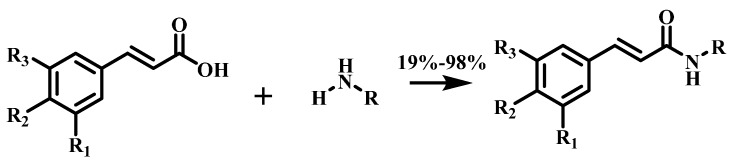
General procedure for the synthesis of cinnamide derivatives. Reagents and conditions: PyBOP, DMF, Et_3_N, CH_2_Cl_2_, 0 °C (0.5 h) then room temperature (overnight).

Cinnamide derivatives were synthesized using phosphonium salts as condensing reagents by a procedure adapted from Okombi et al. [37]. The synthesis protocol was modified by replacing benzotriazol-1-yloxytris(dimethylamino)-phosphonium hexafluorophosphate (BOP) by benzotriazol-1-yloxytri(pyrrolidino)-phosphonium hexafluorophosphate (PyBOP) [38] to avoid the formation of the carcinogenic side-product hexamethylphosphoric acid triamide (HMPA) [39]. The amide linkage was generated by mixing the free acid with a variety of amines in the presence of PyBOP, triethylamine (Et_3_N) in dimethylformamide (DMF) and CH_2_Cl_2_ (Scheme 1). The by-product tripyrrolidinophosphoric acid triamide (TPPA) derived from PyBOP was more viscous and less polar than HMPA. Furthermore, its boiling point was higher, making the separation of the cinnamide derivative and TPPA more difficult and leading to low yields [39]. Cinnamide derivatives (Table 1) were obtained with yields ranging from 19% to 98%.

(*E*)-*N*-(2-phenylethyl)-3,4-dihydroxycinnamide (compound **1**, CAPA) was chosen as the basic structure (Table 1). Subsequently, compounds **2**–**15** were synthesized to explore the effect on arginase inhibition of hydroxyl groups at different positions on the two aromatic rings and also their substitutions by methyl groups. Compounds **16** and **17** were synthesized to help explain contribution of the double bond of caffeoyl moiety and of the single bond of the phenethylamine moiety. Compound **18** was synthesized to explore the effect of a methylcarboxylate group on the phenethylamine moiety. Finally, to evaluate the importance of the aromatic ring on the phenethylamine moiety, it was replaced by a sulfhydryl group (compound **19**). The structures of these compounds were confirmed by NMR, HRMS (ESI), IR spectrum, and by comparison with previous published data.

### 2.2. Arginase Inhibitory Activity

Arginase inhibitory activity was first evaluated using an in vitro assay optimizing the protocol of Corraliza et al. (1994) [40] by miniaturization and by using commercially-available purified bovine arginase (b-ARG I) instead of animal tissue [34]. The most active compound was then evaluated on a recombinant human arginase (h-ARG I). *N*^ω^-hydroxy-nor-l-arginine (nor-NOHA) and CGA served as reference inhibitors.

**Figure 2:**
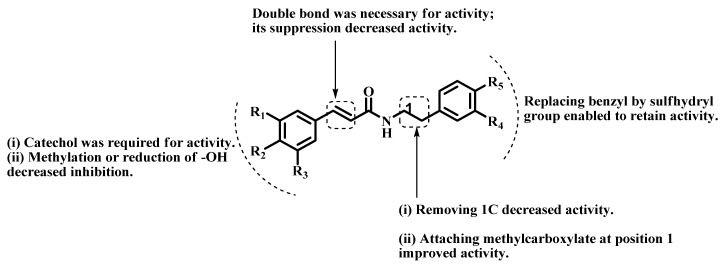
Structure–activity relationships for cinnamide derivatives on b-ARG I.

**Figure 3:**
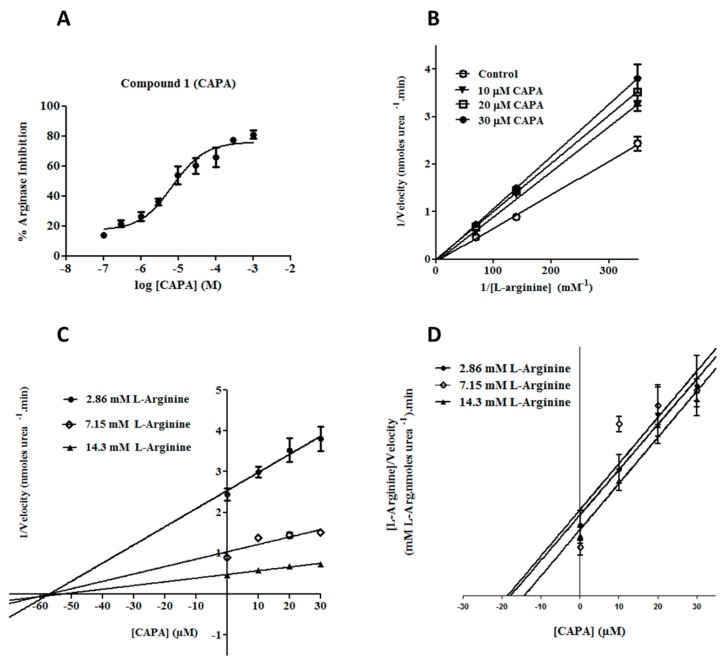
Kinetics of arginase inhibition by **1** (CAPA). The IC_50_ was determined from a sigmoidal curve (**A**). The competitive inhibition mechanism was determined by analysis of the: Lineweaver–Burk (**B**); Dixon (**C**); and Cornish–Bowden (**D**) plots. Each data point represents the mean of three in-triplicate independent experiments.

As shown in Table 1, arginase inhibition on b-ARG I was higher than 50% for eleven target compounds at 100 μM. IC_50_ values of these eleven compounds were determined to better estimate their potencies and discuss the structure–activity relationships. It is worth noting that all eleven of these compounds contain a catechol group, on the cinnamoyl (compounds **1**, **6**, **16** and **19**), benzoyl (compound **17**) or phenethyl parts (compounds **12**–**15**), or on both parts (compounds **11** and **18**). In fact, suppression or methylation of one or two hydroxyl groups of the catechol function led to a severe decrease in arginase inhibition activity (compound **1** vs. **2**–**5**). Our main result is that structural modifications led to an improvement of the activity for compound **1**, caffeic acid phenylamide (CAPA), which displayed a lower IC_50_ value (6.9 ± 1.3 μM, Figure 3A) compared to our natural reference compound CGA (IC_50_ = 10.6 ± 1.6 µM). Nevertheless, this value still remains higher than that of the reference compound nor-NOHA. In terms of structure–activity relationships (SARs), functionalization of the phenethyl part of **1** by one (compound **6**) or two (compound **11**) hydroxyl groups decreased the activity. However, if the cinnamoyl part remained unsubstituted, inhibition was partially restored by the presence of a catechol group on the phenethyl part (compound **15** vs. **11**). This result confirmed the important role played by the catechol function for the arginase inhibitory activity of this series of compounds. Reducing the length of the molecule by suppressing a bond in either the caffeoyl side or the phenethyl side also decreased activity (compound **6** vs. **16**, **17**). However, retaining most or all of the cinnamoyl part seemed to be of greater importance than that of the phenethyl part. Concerning the single bond of the phenylethylamine moiety, a preliminary investigation has been made involving the substitution of the ethyl linker by a methylcarboxylate group (**18**) and by replacing the aromatic ring by a sulfhydryl group (**19**). The methylcarboxylate group at position 1 of the phenethylamine moiety improved the inhibitory activity (compound **18** vs. **11**), and replacing the aromatic ring of the phenethylamine moiety by the sulfhydryl group had little effect on the inhibitory activity (compound **18** vs. **19**), suggesting that no specific interaction occurred between this part of the molecule and the mouth of the active site (Figure 2). Therefore, pharmacomodulations in this position could be expected to increase compounds affinity toward the active site.

Considering its effect on b-ARG I, CAPA (**1**) was evaluated on a recombinant h-ARG I. The reference inhibitor nor-NOHA was previously evaluated on this model and gave an IC_50_ value of 5.7± 0.6 µM. The IC_50_ value of CAPA was found to be 60.3 ± 7.8 µM. This value is higher than that determined on b-ARG I (IC_50_ = 6.9 ± 1.3 µM). These results illustrated the fact that the evaluation on b-ARG I as an easy-to-handle and cheap source of mammalian arginase could be used in a preliminary study in order to search new potential active compounds. However, an evaluation of the most active compounds on h-ARG I is required before further in vivo studies.

### 2.3. Enzyme Kinetic Studies for CAPA (**1**)

The mechanism of inhibition and the inhibition constant (K_i_) of compound **1** (CAPA) was assessed by enzyme-kinetic studies. The kinetic data were used to generate the Lineweaver–Burk, Dixon and Cornish–Bowden plots (Figure 3B–D), showing that CAPA is a competitive inhibitor of arginase [41].

The K_i_ values for CAPA, CGA and nor-NOHA could be calculated on the basis of the Cheng–Prusoff equation [K_i_ = IC_50_/(1 + [S]/K_m_)] [42], with [S] = 14.3 mM of l-arginine and the Michaelis–Menten constant of b-ARG I K_m_ = 55.5 ± 10.5 µM [34] (Table 2).

The in vitro affinity of CAPA was better than that of CGA (K_i_ 5.5 ± 1.0 vs. 8.4 ± 1.2 µM, respectively). In the last few years, several chemical groups that target the bimanganese cluster have been identified. Here, the in vitro affinity of CAPA was similar to that described for amino imidazole derivative [28], and better than thiosemicarbazide [43], sulfamide [27], nitro [29] and aldehyde [30] derivatives, which displayed Ki greater than 50 µM. Cinnamides could be added to this list and may help to design future inhibitors.

### 2.4. Molecular Docking Studies

In order to gain further insight into the inhibitory mechanism, the binding modes of CAPA (**1**) and CGA were determined by docking simulations on h-ARG I (pdb id: 3kv2). The structure of b-ARG I is not available in the Protein Data Bank but present 100% homology with h-ARG I in the active site. In addition, the alignment of the b-ARG I and h-ARG I sequences using the stand-alone Java Web Start application, accessible from the PDB server (http://www.rcsb.org), revealed a high level of similarity between the two sequences (91% and 95%, respectively). A few residues were different, but not involved in the active site of the two arginases, whose residues are strictly conserved. The most active compound **1** (CAPA), CGA and nor-NOHA were successfully docked into the active pocket of h-ARG I by using a FlexX docking program implemented in LeadIt 2.0.2 software [44].

**Figure 4:**
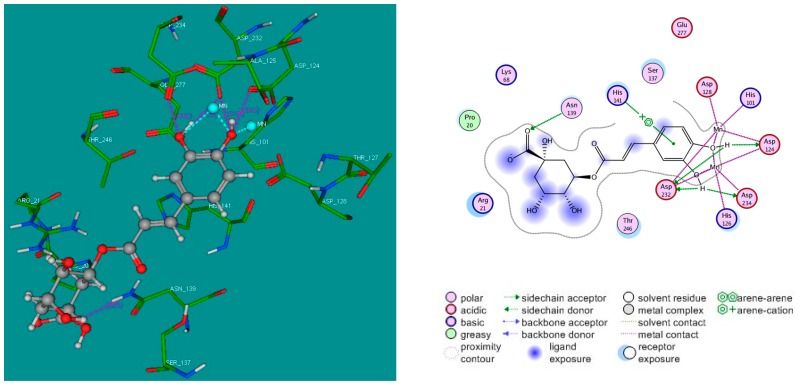
3D (**left**); and 2D (**right**) binding modes of CGA inside the active site of h-ARG I. For the 3D binding mode, CGA is represented as a “ball and stick” form: C (**grey**), O (**red**), and H (**white**); manganese ions are represented as cyan spheres; and the crucial residues of the binding site are represented by lines. H-bonds are represented as pink dotted lines, while the metal coordination bonds are represented by cyan dotted lines. Docking simulations were performed by FlexX program implemented in LeadIt 2.0.2 software, and the pictures of docking solution were created by MOE 2008.10 software.

The results obtained with CGA confirmed that the quinoyl moiety interact with residues of the mouth of the pocket formed by the catalytic site, helping the caffeoyl moiety of chlorogenic acid to approach the bottom of the active site and interact with Asp124, Asp232, Asp234 by H-interactions and His141 via cation–π interaction (Figure 4). Interestingly, the catechol group could chelate the Mn^2+^ ions in the active site of arginase.

**Figure 5:**
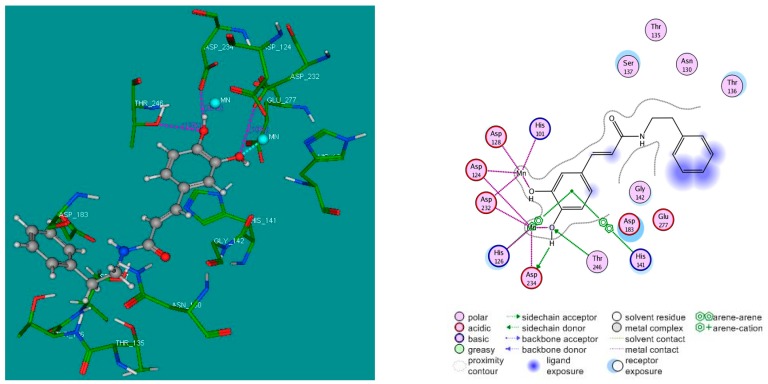
3D (**left**); and 2D (**right**) binding modes of CAPA (**1**) inside the active site of h-ARG I. For the 3D binding mode, CAPA is represented as a “ball and stick” form: C (**grey**), O (**red**), and H (**white**); manganese ions are represented as cyan spheres; and the crucial residues of the binding site are represented by lines. H-bonds are represented as pink dotted lines, while the metal coordination bonds are represented by cyan dotted lines. Docking simulations were performed by FlexX program implemented in LeadIt 2.0.2 software, and the pictures of docking solution were created by MOE 2008.10 software.

Since CAPA was identified as a potential lead compound of the series, interactions of this compound with the residues of the active site of the enzyme were examined more closely. We noted with interest that the caffeoyl moiety of CAPA is able to interact with the Mn^2+^ cofactor and can make hydrogen interactions with Asp234 and Thr246 as well as π–π interactions with His141 and His126 of h-ARG I active site. The phenethyl moiety could have hydrophobic interactions residues at the mouth of active pocket (Figure 5).

In addition, it is worth noting that compound **15** took a position that was opposite to that of CAPA, the cinnamoyl moiety staying at the mouth whereas the phenethylamine with a catechol group approached the active pocket of b-ARG I (data not shown). These opposite binding modes confirmed the hypothesis of the crucial role played by the catechol group in arginase inhibition, and clearly explained why the activity was recovered by compound **15** despite the fact the cinnamoyl moiety was unfunctionalized.

## 3. Materials and Methods

### 3.1. Chemistry

All reagents were purchased from Sigma-Aldrich and used without further purification, except for the purified liver bovine arginase I (b-ARG I) which was from MP Biomedicals (One unit (1 U) of b-ARG I is defined by this manufacturer as the amount of enzyme that converted 1 µmole of l-arginine to urea and l-ornithine per minute at pH 9.5 and 37 °C), and for the recombinant human arginase I (h-ARG I) (BXC572/n P0408M10) which was from Interchim (Number of units is not defined by the manufacturer and thus a preliminary study (data not shown) allowed us to choose the concentration of enzyme leading to an absorbance of about 1). Solvents (methanol, ethyl acetate, cyclohexane and dichloromethane) were supplied by Carlo Erba Reagents and VWR Chemicals companies. Reactions were monitored by thin-layer chromatography (TLC) on pre-coated silica gel aluminum plates (Macherey-Nagel) and visualized under UV light (254 and 365 nm). Flash column chromatography was carried out using EasyVarioFlash^®^ DL cartridges (Merck, Fontenay-sous-Bois, France) for dry loading, eluting with CH_2_Cl_2_ and MeOH. ^1^H-NMR at 300 MHz and ^13^C-NMR at 75 MHz (only for compounds lacking analytical data from literature) were acquired using a Bruker AC300 spectrometer (Bruker BioSpin, Wissembourg, France). Chemical shifts (*δ*) were reported in parts per million (ppm) relative to the residual solvent signals. Coupling constants (*J*) were reported in hertz (Hz). Data were presented as follows: chemical shift (*δ*, ppm), multiplicity (s, singlet; bs, broad singlet; d, doublet; dd, doublet of doublet; t, triplet; q, quadruplet; m, multiplet), coupling constant (*J*, Hz), and integration. High-resolution electrospray ionisation mass spectra (HRMS-ESI) were analyzed by using the SCA Illkirch QToF instrument.

### 3.2. General Procedure for the Synthesis of Amide Derivatives ***1**−**19***

A solution of substituted cinnamic acid or 3,4-dihydroxybenzoic acid (1.66 mmol) and trimethylamine (Et_3_N) (0.35 mL, 2.49 mmol, 1.5 equivalent (eq)) in dimethylformamide (DMF) (3.5 mL) was cooled in an ice bath. A corresponding amine (1.66 mmol, 1 eq) was added and followed by addition of a solution of benzotriazolyloxy-tris(pyrrolidino)-phosphonium hexafluorophosphate (PyBOP) (864 mg, 1.66 mmol, 1 eq) in dichloromethane (CH_2_Cl_2_) (3.5 mL). The mixture was stirred for 30 min at 0 °C and left overnight at room temperature before CH_2_Cl_2_ was evaporated under vacuum. Then, 30 mL of water was added to the remaining solution and the resulting mixture was extracted with ethyl acetate (EtOAc) (3 × 75 mL). The organic phase was successively washed with 100 mL of 1 M HCl solution, 100 mL of water, 100 mL of 1 M NaHCO_3_ solution and 100 mL of brine, then dried over Na_2_SO_4_, filtered and evaporated under vacuum. The crude product was purified by silica gel flash chromatography (eluent: CH_2_Cl_2_/MeOH 98:2 to 80:20) to afford the desired compound [37].

*(E)-N-(2-Phenylethyl)-3,4-dihydroxycinnamide* (**1**). General procedure using 3,4-dihydroxycinnamic acid (300 mg, 1.66 mmol) and 2-phenylethylamine (209 µL, 1.66 mmol) afforded compound **1** (213 mg, 45%) as a white solid. *R_f_* = 0.2 (CH_2_Cl_2_/MeOH 95:5); IR (ATR) γ cm^−1^: 3259, 3087, 3061, 3027, 2969, 2935, 2870, 1702, 1652, 1590, 1513; ^1^H-NMR (Acetone-*d*_6_, 300 MHz): δ 8.23 (s, 2H), 7.41 (d, *J* = 15.6 Hz, 1H), 7.31–7.17 (m, 6H), 7.06 (s, 1H), 6.93 (d, *J* = 8.0 Hz, 1H), 6.83 (d, *J* = 8.0 Hz, 1H), 6.43 (d, *J* = 15.6 Hz, 1H), 3.57–3.50 (m, 2H), 2.90–2.82 (m, 2H); HRMS (ESI) *m*/*z* calcd for C_17_H_18_NO_3_ [M + H]^+^: 284.1208, found: 284.1288. The analytical data were in line with previously described data [45].

*(E)-N-(2-Phenylethyl)-3-methoxy-4-hydroxycinnamide* (**2**). General procedure using 3-methoxy-4-hydroxycinnamic acid (322 mg, 1.66 mmol) and 2-phenylethylamine (209 µL, 1.66 mmol) afforded compound **2** (388 mg, 79%) as a white solid. *R_f_* = 0.33 (Cyclohexane/EtOAc 4:6); IR (ATR) γ cm^−1^: 3270, 3063, 3027, 2935, 1652, 1585, 1510; ^1^H-NMR (MeOD-*d*_4_, 300 MHz): δ 7.44 (d, *J* = 15.7 Hz, 1H), 7.32–7.17 (m, 5H), 7.12 (s, 1H), 7.03 (d, *J* = 8.4 Hz, 1H), 6.79 (d, *J* = 8.4 Hz, 1H), 6.40 (d, *J* = 15.7 Hz, 1H), 3.88 (s, 3H), 3.52 (t, *J* = 7.2 Hz, 2H), 2.86 (t, *J* = 7.2 Hz, 2H); HRMS (ESI) *m*/*z* calcd for C_18_H_20_NO_3_ [M + H]^+^: 298.1365, found: 298.1442. The analytical data were in line with previously described data [46].

*(E)-N-(2-Phenylethyl)-4-hydroxycinnamide* (**3**). General procedure using 4-hydroxycinnamic acid (272 mg, 1.66 mmol) and 2-phenylethylamine (209 µL, 1.66 mmol) afforded compound **3** (288 mg, 65%) as a colorless syrup. *R_f_* = 0.56 (CH_2_Cl_2_/MeOH 95:5); IR (ATR) γ cm^−1^: 3400, 3263, 3085, 3064, 3026, 2934, 2813, 2750, 2687, 2608, 1651, 1600, 1579, 1511; ^1^H-NMR (MeOD-*d*_4_, 300 MHz): δ 7.47–7.38 (m, 3H), 7.31–7.17 (m, 5H), 6.78 (d, *J* = 8.4 Hz, 2H), 6.38 (d, *J* = 15.7 Hz, 1H), 3.51 (t, *J* = 7.2 Hz, 2H), 2.85 (t, *J* = 7.2 Hz, 2H); HRMS (ESI) *m*/*z* calcd for C_17_H_18_NO_2_ [M + H]^+^: 268.1259, found: 268.1336. The analytical data were in line with previously described data [47].

*(E)-N-(2-Phenylethyl)-3,4,5-trimethoxycinnamide* (**4**). General procedure using 3,4,5-trimethoxycinnamic acid (395 mg, 1.66 mmol) and 2-phenylethylamine (209 µL, 1.66 mmol) afforded compound **4** (511 mg, 90%) as a white solid. *R_f_* = 0.56 (CH_2_Cl_2_/MeOH 95:5); IR (ATR) γ cm^−1^: 3305, 3087, 3062, 3030, 3005, 2967, 2933, 2862, 2835, 1653, 1615, 1581, 1536, 1506; ^1^H-NMR (CDCl_3_, 300 MHz): δ 7.53 (d, *J* = 15.5 Hz, 1H), 7.36–7.22 (m, 5H), 6.71 (s, 2H), 6.22 (d, *J* = 15.5 Hz, 1H), 5.55 (bs, 1H), 3.88 (s, 6H), 3.87 (s, 3H), 3.71–3.65 (m, 2H), 2.89 (t, *J* = 6.8 Hz, 2H); ^13^C-NMR (CDCl_3_, 75 MHz): *δ* 165.9, 153.5, 141.1, 139.6, 138.9, 130.5, 128.9, 128.8, 126.7, 120.0, 104.9, 61.1, 56.2, 40.9, 35.7; HRMS (ESI) *m*/*z* calcd for C_20_H_24_NO_4_ [M + H]^+^: 342.1627, found: 342.1709.

*(E)-N-(2-Phenylethyl)cinnamide* (**5**). General procedure using cinnamic acid (246 mg, 1.66 mmol) and 2-phenylethylamine (209 µL, 1.66 mmol) afforded compound **5** (330 mg, 79%) as a white solid. *R_f_* = 0.7 (CH_2_Cl_2_/MeOH 95:5); IR (ATR) γ cm^−1^: 3265, 3062, 3030, 2971, 2944, 2862, 1959, 1895, 1819, 1660, 1651, 1603, 1579, 1544; ^1^H-NMR (CDCl_3_, 300 MHz): δ 7.62 (d, *J* = 15.5 Hz, 1H), 7.50–7.47 (m, 2H), 7.40–7.31 (m, 5H), 7.25–7.22 (m, 3H), 6.32 (d, *J* = 15.5 Hz, 1H), 5.58 (bs, 1H), 3.71–3.65 (m, 2H), 2.90 (t, *J* = 6.6, 2H); HRMS (ESI) *m*/*z* calcd for C_17_H_18_NO [M + H]^+^: 252.1310, found : 252.1386. The analytical data were in line with previously described data [48].

*(E)-N-(2-(4-Hydroxyphenyl)ethyl)-3,4-dihydroxycinnamide* (**6**). General procedure using 3,4-dihydroxycinnamic acid (300 mg, 1.66 mmol) and 2-(4-hydroxyphenyl)ethylamine (228 mg, 1.66 mmol) afforded compound **6** (358 mg, 72%) as a slight yellow solid. *R_f_* = 0.64 (Cyclohexane/EtOAc 1:9); IR (ATR) γ cm^−1^: 3349, 3167, 3030, 2960, 2930, 2877, 1727, 1645, 1602, 1578, 1535, 1514; ^1^H-NMR (DMSO-*d*_6_, 300 MHz): δ 9.23 (bs, 3H), 8.02 (bs, 1H), 7.21 (d, *J* = 15.6 Hz, 1H), 7.0 (d, *J* = 8.2 Hz, 2H), 6.93 (s, 1H), 6.82 (d, *J* = 8.0 Hz, 1H), 6.73 (d, *J* = 8.0 Hz, 1H), 6.67 (d, *J* = 8.2 Hz, 2H), 6.31 (d, *J* = 15.6 Hz, 1H), 3.34–3.28 (m, 2H), 2.63 (t, *J* = 7.3 Hz, 2H); HRMS (ESI) *m*/*z* calcd for C_17_H_18_NO_4_ [M + H]^+^: 300.1236, found: 300.1235. The analytical data were in line with previously described data [49].

*(E)-N-(4-Hydroxyphenethyl)-3-methoxy-4-hydroxycinnamide* (**7**). General procedure using 3-methoxy-4-hydroxycinnamic acid (322 mg, 1.66 mmol) and 4-hydroxyphenethylamine (228 mg, 1.66 mmol) afforded compound **7** (298 mg, 57%) as a white solid. *R_f_* = 0.22 (Cyclohexane/EtOAc 5:5); IR (ATR) γ cm^−1^: 3287, 3015, 2936, 1651, 1586, 1510; ^1^H-NMR (MeOD-*d*_4_, 300 MHz): δ 7.43 (d, *J* = 16.0 Hz, 1H), 7.12 (s, 1H), 7.07–7.01 (m, 3H), 6.79 (d, *J* = 8.1 Hz, 1H), 6.71 (d, *J* = 8.0 Hz, 2H), 6.40 (d, *J* = 16.0 Hz, 1H), 3.88 (s, 3H), 3.47 (t, *J* = 6.9 Hz, 2H), 2.76 (t, *J* = 6.9 Hz, 2H); HRMS (ESI) *m*/*z* calcd for C_18_H_20_NO_4_ [M + H]^+^: 314.1314, found: 314.1396. The analytical data were in line with previously described data [50].

*(E)-N-(4-Hydroxyphenethyl)-4-hydroxycinnamide* (**8**). General procedure using 4-hydroxycinnamic acid (272 mg, 1.66 mmol) and 4-hydroxyphenethylamine (228 mg, 1.66 mmol) afforded compound **8** (233 mg, 50%) as a white solid. *R_f_* = 0.30 (Cyclohexane/EtOAc 5:5); IR (ATR) γ cm^−1^: 3431, 3171, 3024, 2942, 1895, 1660, 1622, 1602, 1590, 1530, 1510; ^1^H-NMR (MeOD-*d*_4_, 300 MHz): δ 7.46–7.38 (m, 3H), 7.05 (d, *J* = 8.1 Hz, 2H), 6.78 (d, *J* = 8.3 Hz, 2H), 6.71 (d, *J* = 8.1 Hz, 2H), 6.38 (d, *J* = 15.6 Hz, 1H), 3.45 (t, *J* = 7.3 Hz, 2H), 2.75 (t, *J* = 7.3 Hz, 2H); HRMS (ESI) *m*/*z* calcd for C_17_H_18_NO_3_ [M + H]^+^: 284.1208, found: 284.1285. The analytical data were in line with previously described data [51].

*(E)-N-(4-Hydroxyphenethyl)-3,4,5-trimethoxycinnamide* (**9**). General procedure using 3,4,5-trimethoxycinnamic acid (395 mg, 1.66 mmol) and 4-hydroxyphenethylamine (228 mg, 1.66 mmol) afforded compound **9** (586 mg, 98%) as a white solid. *R_f_* = 0.26 (Cyclohexane/EtOAc 5:5); IR (ATR) γ cm^−1^: 3323, 3012, 2963, 2940, 2909, 2888, 2847, 2824, 2605, 1994, 1652, 1605, 1580, 1544, 1516, 1504; ^1^H-NMR (MeOD-*d*_4_, 300 MHz): δ 7.44 (d, *J* = 15.7 Hz, 1H), 7.06 (d, *J* = 8.1 Hz, 2H), 6.86 (s, 2H), 6.72 (d, *J* = 8.1 Hz, 2H), 6.51 (d, *J* = 15.7 Hz, 1H), 3.87 (s, 6H), 3.78 (s, 3H), 3.48 (t, *J* = 7.2 Hz, 2H), 2.76 (t, *J* = 7.2 Hz, 2H); ^13^C-NMR (MeOD-*d*_4_ + 10% CDCl_3_, 75 MHz): δ 168.5, 156.8, 154.5, 141.6, 140.4, 132.1, 131.1, 130.7, 121.2, 116.2, 106.1, 61.3, 56.7, 42.5, 35.7; HRMS (ESI) *m*/*z* calcd for C_20_H_24_NO_5_ [M + H]^+^: 358.1576, found: 358.1657.

*(E)-N-(4-Hydroxyphenethyl)-cinnamide* (**10**). General procedure using cinnamic acid (246 mg, 1.66 mmol) and 4-hydroxyphenethylamine (228 mg, 1.66 mmol) afforded compound **10** (359 mg, 81%) as a white solid. *R_f_* = 0.44 (Cyclohexane/EtOAc 5:5); IR (ATR) γ cm^−1^: 3429, 3268, 3059, 3022, 2941, 2922, 2858, 1889, 1665, 1624, 1611, 1592, 1532, 1512; ^1^H-NMR (MeOD-*d*_4_, 300 MHz): δ 7.56–7.49 (m, 3H), 7.42–7.33 (m, 3H), 7.06 (d, *J* = 8.2 Hz, 2H), 6.72 (d, *J* = 8.2 Hz, 2H), 6.57 (d, *J* = 16.0 Hz, 1H), 3.47 (t, *J* = 6.9 Hz, 2H), 2.76 (t, *J* = 6.9 Hz, 2H); HRMS (ESI) *m*/*z* calcd for C_17_H_18_NO_2_ [M + H]^+^: 268.1259, found: 268.1337. The analytical data were in line with previously described data [52].

*(E)-N-(3,4-Dihydroxyphenethyl)-3,4-dihydroxycinnamide* (**11**). General procedure using 3,4-dihydroxycinnamic acid (300 mg, 1.66 mmol) and 3,4-dihydroxyphenethylamine hydrochloride (315 mg, 1.66 mmol) afforded compound **11** (229 mg, 44%) as a slight yellow solid. *R_f_* = 0.45 (CH_2_Cl_2_/MeOH 85:15); IR (ATR) γ cm^−1^: 3214, 2938, 2723, 1695, 1650, 1586, 1514; ^1^H-NMR (Acetone-*d*_6_, 300 MHz): δ 8.15 (bs, 4H), 7.43 (s, 1H), 7.42 (d, *J* = 15.6 Hz, 1H), 7.06 (s, 1H), 6.89 (d, *J* = 8.1 Hz, 1H), 6.8 (d, *J* = 8.2 Hz, 1H), 6.72 (s, 1H), 6.69 (d, *J* = 8.2 Hz, 1H), 6.52 (d, *J* = 8.1 Hz, 1H), 6.44 (d, *J* = 15.6 Hz, 1H), 3.47 (m, 2H), 2.67 (t, *J* = 6.9 Hz, 2H); HRMS (ESI) *m*/*z* calcd for C_17_H_18_NO_5_ [M + H]^+^: 316.1185; found: 316.1181. The analytical data were in line with previously described data [53].

*(E)-N-(3,4-Dihydroxyphenethyl)-3-methoxy-4-hydroxycinnamide* (**12**). General procedure using 3-methoxy-4-hydroxycinnamic acid (322 mg, 1.66 mmol) and 3,4-dihydroxyphenethylamine hydrochloride (315 mg, 1.66 mmol) afforded compound **12** (164 mg, 30%) as a white solid. *R_f_* = 0.14 (CH_2_Cl_2_/MeOH 95:5); IR (ATR) γ cm^−1^: 3321, 2969, 2937, 2841, 2720, 1651, 1585, 1510; ^1^H-NMR (MeOD-*d*_4_, 300 MHz): δ 7.43 (d, *J* = 16.0 Hz, 1H), 7.12 (s, 1H), 7.02 (d, *J* = 8.0 Hz, 1H), 6.79 (d, *J* = 8.3 Hz, 1H), 6.71–6.67 (m, 2H), 6.55 (d, *J* = 8.0 Hz, 1H), 6.40 (d, *J* = 16.0 Hz, 1H), 3.88 (s, 3H), 3.46 (t, *J* = 7.0 Hz, 2H), 2.70 (t, *J* = 7.0 Hz, 2H). ^13^C-NMR (MeOD-*d*_4_, 75 MHz): *δ* 169.3, 149.9, 149.4, 146.4, 144.9, 142.2, 132.2, 128.4, 123.4, 121.2, 118.9, 117.0, 116.6, 116.5, 111.6, 56.5, 42.7, 36.2; HRMS (ESI) *m*/*z* calcd for C_18_H_20_NO_5_ [M + H]^+^: 330.1263; found: 330.1342.

*(E)-N-(3,4-Dihydroxyphenethyl)-4-hydroxycinnamide* (**13**). General procedure using 4-hydroxycinnamic acid (272 mg, 1.66 mmol) and 3,4-dihydroxyphenethylamine hydrochloride (315 mg, 1.66 mmol) afforded compound **13** (204 mg, 41%) as a white solid. *R_f_* = 0.15 (CH_2_Cl_2_/MeOH 95:5); IR (ATR) γ cm^−1^: 3270, 3025, 2938, 2817, 2701, 1696, 1648, 1577, 1511; ^1^H-NMR (MeOD-*d*_4_, 300 MHz): δ 7.44 (d, *J* = 16.0 Hz, 1H), 7.40 (d, *J* = 8.4 Hz, 2H), 6.79 (d, *J* = 8.4 Hz, 2H), 6.70–6.67 (m, 2H), 6.55 (d, *J* = 8.0 Hz, 1H), 6.38 (d, *J* = 16.0 Hz, 1H), 3.45 (t, *J* = 7.0 Hz, 2H), 2.70 (t, *J* = 7.0 Hz, 2H); HRMS (ESI) *m*/*z* calcd for C_17_H_18_NO_4_ [M + H]^+^: 300.1158; found: 300.1233. The analytical data were in line with previously described data [54].

*(E)-N-(3,4-Dihydroxyphenethyl)-3,4,5-trimethoxycinnamide* (**14**). General procedure using 3,4,5-trimethoxycinnamic acid (395 mg, 1.66 mmol) and 3,4-dihydroxyphenethylamine hydrochloride (315 mg, 1.66 mmol) afforded compound **14** (436 mg, 70%) as a white solid. *R_f_* = 0.26 (CH_2_Cl_2_/MeOH 95:5); IR (ATR) γ cm^−1^: 3337, 3105, 3040, 2963, 2944, 2843, 2728, 1706, 1658, 1613, 1605, 1585, 1544, 1504; ^1^H-NMR (MeOD-*d*_4_, 300 MHz): δ 7.44 (d, *J* = 15.7 Hz, 1H), 6.87 (s, 2H), 6.71–6.67 (m, 2H), 6.57–6.48 (m, 2H), 3.87 (s, 6H), 3.78 (s, 3H), 3.47 (t, *J* = 7.2 Hz, 2H), 2.71 (t, *J* = 7.2 Hz, 2H); HRMS (ESI) *m*/*z* calcd for C_20_H_24_NO_6_ [M + H]^+^: 374.1525; found: 374.1605. The analytical data were in line with previously described data [55].

*(E)-N-(3,4-Dihydroxyphenethyl)cinnamide* (**15**). General procedure using cinnamic acid (246 mg, 1.66 mmol) and 3,4-dihydroxyphenethylamine hydrochloride (315 mg, 1.66 mmol) afforded compound **15** (293 mg, 62%) as a colorless syrup. *R_f_* = 0.29 (CH_2_Cl_2_/MeOH 95:5); IR (ATR) γ cm^−1^: 3265, 2927, 1702, 1655, 1599, 1519; ^1^H-NMR (MeOD-*d*_4_, 300 MHz): δ 7.56–7.49 (m, 3H), 7.42–7.35 (m, 3H), 6.71–6.67 (m, 2H), 6.60–6.54 (m, 2H), 3.47 (t, *J* = 7.2 Hz, 2H), 2.71 (t, *J* = 7.2 Hz, 2H); HRMS (ESI) *m*/*z* calcd for C_17_H_18_NO_3_ [M + H]^+^: 284.1208; found: 284.1283. The analytical data were in line with previously described data [54].

*(E)-N-(4-Hydroxyphenyl)-3,4-dihydroxycinnamide* (**16**). General procedure using 3,4-dihydroxycinnamic acid (300 mg, 1.66 mmol) and 4-hydroxyphenylamine (181 mg, 1.66 mmol) afforded compound **16** (84 mg, 19%) as a brown solid. *R_f_* = 0.65 (Cyclohexane/EtOAc 1:9); IR (ATR) γ cm^−1^: 3210, 1696, 1648, 1588, 1535, 1508; ^1^H-NMR (DMSO-*d*_6_, 300 MHz): δ 9.83 (s, 1H), 9.46 (bs, 1H), 9.21 (bs, 2H), 7.46 (d, *J* = 8.6 Hz, 2H), 7.33 (d, *J* = 15.6 Hz, 1H), 6.98 (s, 1H), 6.88 (d, *J* = 8.3 Hz, 1H), 6.76 (d, *J* = 8.3 Hz, 1H), 6.70 (d, *J* = 8.6 Hz, 2H), 6.49 (d, *J* = 15.6 Hz, 1H); HRMS (ESI) *m*/*z* calcd for C_15_H_14_NO_4_ [M + H]^+^: 272.0923, found: 272.0918. The analytical data were in line with previously described data [56].

*N-(4-Hydoxyphenethyl)-3,4-dihydroxybenzamide* (**17**). General procedure using 3,4-dihydroxybenzoic acid (256 mg, 1.66 mmol) and 4-hydroxyphenethylamine (228 mg, 1.66 mmol) afforded compound **19** (53 mg, 12%) as a colorless syrup. *R_f_* = 0.19 (CH_2_Cl_2_/MeOH 95:5); IR (ATR) γ cm^−1^: 3177, 2970, 2948, 2869, 1698, 1632, 1589, 1553, 1510; ^1^H-NMR (MeOD-*d*_4_, 300 MHz): δ 7.24 (d, *J* = 2.0 Hz, 1H), 7.15 (dd, *J*_1_ = 8.2 Hz, *J*_2_ = 2.0 Hz, 2H), 7.06 (d, *J* = 8.3 Hz, 2H), 6.78 (d, *J* = 8.2 Hz, 1H), 6.71 (d, *J* = 8.3 Hz, 2H), 3.49 (t, *J* = 7.3 Hz, 2H), 2.78 (t, *J* = 7.3 Hz, 2H). HRMS (ESI) *m*/*z* calcd for C_15_H_16_NO_4_ [M + H]^+^: 274.1001, found: 274.1072. The analytical data were in line with previously described data [57].

*3-(3,4-Dihydroxyphenyl)-2-[3-(3,4-dihydroxyphenyl)-acryloylamino]-propionic acid methyl ester* (**18**). General procedure using 3,4-dihydroxycinnamic acid (300 mg, 1.66 mmol) and l-3,4-dihydroxyphenylalanine methyl ester (315 mg, 1.66 mmol, previously prepared following [58]) afforded compound **18** (332 mg, 54%) as a yellow solid. *R_f_* = 0.5 (CH_2_Cl_2_/MeOH 85:15); IR (ATR) γ cm^−1^: 3308, 2953, 2838, 2725, 1722, 1650, 1594, 1514; ^1^H-NMR (DMSO-*d*_6_, 300 MHz): δ 8.35 (d, *J* = 6.9 Hz, 1H), 7.20 (d, *J* = 15.6 Hz, 1H), 6.93 (s, 1H), 6.83 (d, *J* = 8.1 Hz, 1H), 6.73 (d, *J* = 8.1 Hz, 1H), 6.61–6.59 (m, 2H), 6.45 (d, *J* = 8.1 Hz, 1H), 6.38 (d, *J* = 15.7 Hz, 1H), 4.46 (m, 1H), 3.60 (s, 3H), 2.89–2.72 (m, 2H); HRMS (ESI) *m*/*z* calcd for C_19_H_20_NO_7_ [M + H]^+^: 374.1240; found: 374.1240. The analytical data were in line with previously described data [59].

*(E)-N-(3,4-Dihydroxycinnamoyl)-l-cysteine methyl ester* (**19**). General procedure using 3,4-dihydroxycinnamic acid (300 mg, 1.66 mmol) and l-cysteine methyl ester hydrochloride (285 mg, 1.66 mmol) afforded compound **19** ( 50.8 mg, 10%) as a white solid. *R_f_* = 0.19 (CH_2_Cl_2_/MeOH 95:5); IR (ATR) γ cm^−1^: 3472, 3400, 3318, 3163, 2952, 2551, 1730, 1654, 1592, 1524; ^1^H-NMR (Acetone-*d*_6_, 300 MHz): δ 8.31 (bs, 2H), 7.52 (d, *J* = 7.6 Hz, 1H), 7.44 (d, *J* = 15.6 Hz, 1H), 7.09 (d, *J* = 1.8 Hz, 1H), 6.97 (dd, *J*_1_ = 1.8 Hz, *J*_2_ = 8.2 Hz, 1H), 6.84 (d, *J* = 8.2 Hz, 1H), 6.62 (d, *J* = 15.6 Hz, 1H), 4.87–4.80 (m, 1H), 3.73 (s, 3H), 3.05–2.93 (m, 2H), 1.96 (t, *J* = 8.5 Hz, 1H); HRMS (ESI) *m*/*z* calcd for C_13_H_16_NO_5_S [M + H]^+^: 298.0671; found: 298.0750. The analytical data were in line with previously described data [53].

### 3.3. Biological Evaluation

#### 3.3.1. In Vitro Enzymatic Assay

Arginase catalyzes the hydrolysis of l-arginine into l-ornithine and urea. Urea amount produced by this reaction can be detected using a colorimetric assay. We adapted the Corraliza et al. (1994) method [40] by miniaturizing this colorimetric assay and by using a purified bovine liver arginase or a recombinant human arginase I instead of cell lysates [34], as described below. In each well of a microplate, the following solutions were added in this order: (1) 10 µL of a buffer containing and Tris-HCl (50 mM, pH 7.5) 0.1% of Bovine Serum Albumin (TBSA buffer), with or without (control) arginase (0.025 U/µL for b-ARGI or 3.125 µg/mL for h-ARGI); (2) 30 µL of Tris-HCl solution (50 mM, pH 7.5) containing MnCl_2_ 10 mM as a co-factor; (3) 10 µL of a solution containing an inhibitor or its solvent (as a control—see Section 3.3.2); and (4) 20 µL of l-arginine (pH 9.7, 0.05 M). The microplate was covered with a plastic sealing film and incubated for 60 min in a 37 °C water bath. In the case of recombinant h-ARGI the enzyme was activated for 10 min at 55 °C prior to Step (2). The reaction was stopped by adding 120 µL of H_2_SO_4_/H_3_PO_4_/H_2_O (1:3:7) after placing the microplate on ice. Thereafter, 10 µL of alpha-isonitrosopropiophenone (5% in absolute ethanol) was added and the microplate was covered with an aluminum sealing film and heated in an oven at a temperature of 100 °C for 45 min. The microplate was kept in the dark until reading. After 5 min centrifugation and cooling for another 10 min, the microplate was shaken for 2 min and the absorbance was read at 550 nm and 25 °C using a spectrophotometer (Synergy HT BioTeck, Winooski, VT, USA). The level of arginase activity was expressed as relative to the “100% arginase activity” (see Section 3.3.2). Each experimental condition (e.g., various inhibitor concentrations) was repeated three times per microplate.

#### 3.3.2. Determination of IC_50_ Values and Percentages of Arginase Inhibition

For each tested compound, a stock solution (70 mM) was prepared in DMSO and stored at −26 °C. Just before the assay, these stock solutions were successively diluted in ultra-pure water to get the following concentrations: 7000, 2100, 700, 210, 70, 21, 7, 2.1 and 0.7 µM (i.e., final concentrations in the wells of 1000, 300, 100, 30, 10, 3, 1, 0.3, and 0.1 µM, respectively). For a first screening, compounds were only tested at 100 µM (final concentration). Each dilution was incubated with arginase for one hour, as described above. The resulting absorbance was converted into percentage of arginase inhibition, i.e., relative to the absorbance of controls with solvent (“100% arginase activity”), and plotted on a semi-logarithmic scale. The IC_50_ values were estimated with Prism^®^ (GraphPad Software, version 5.0.3, La Jolla, CA, USA) by non-linear sigmoidal curve-fitting.

#### 3.3.3. Enzyme Kinetic Study and Determination of K_i_ Values

The type of inhibition was determined with the same experimental approach with three concentrations of CAPA (10, 20 and 30 μM) and a control under increasing l-arginine concentrations (2.86, 7.15, 14.30 mM). The kinetics data were analyzed using Lineweaver–Burk plot (i.e., 1/velocity vs. 1/[l-arginine] (Figure 2B), Dixon plot (i.e., 1/velocity vs. [inhibitor]) (Figure 2C) and Cornish–Bowden plot (i.e., [l-arginine]/velocity vs. [inhibitor]) (Figure 2D). The type of inhibition was determined following the graphical method described by Cornish–Bowden [41]. The K_i_ values of competitive inhibitors were calculated based on the Cheng–Prusoff equation [K_i_ = IC_50_/(1 + [S]/K_m_)] [42].

### 3.4. Molecular Docking Study

#### 3.4.1. Preparation of Target Protein

The 3D crystal structure of h-ARG I complexed with co-ligand nor-NOHA (pdb id: 3kv2) was retrieved from Protein Data Bank (http://www.rcsb.org). The receptor was prepared following the standard procedure of LeadIt 2.0.2 software. The binding site was defined as all the amino acid residues enclosed within 16.0 Å radius sphere centered by the co-ligand, nor-NOHA. All water molecules were removed.

#### 3.4.2. Preparation of Ligands

Ligands were built and prepared by using Sybyl-X 2.0 software [60]. Hydrogen were added to the 3D structure, which was optimized by energy minimization using Conj Grad Method with convergence criterion set at 0.0001 kcal/(Åmol), max iterations (10,000) and with Gasteiger Hükel charge assigned in Tripos force field; other parameter values were kept at default. Then, the structures were subjected to a simulated annealing run and further optimized to obtain the lowest-energy conformers, which were stored in the database for the docking simulation.

#### 3.4.3. Docking Simulation

The docking simulations were performed using the FlexX docking program [44,61] implemented in LeadIt 2.0.2 software with number of poses set at 10 for analyzing, maximum number of solutions per iteration set at 1000 and maximum number of solutions per fragmentation set at 200, other docking parameters were kept at default setting. The molecular docking technique used in this study was a flexible one in which the ligand binding process was an induced-fit process. The 3D and 2D binding modes of docking solutions were generated by using MOE 2008.10 software [62].

## 4. Conclusions

In summary, a series of nineteen cinnamide derivatives were synthesized and first evaluated for their arginase inhibitory capacities on mammalian arginase I (b-ARG I), together with the two natural caffeic and chlorogenic acids. Among them, chlorogenic acid and six of the synthesized compounds showed an IC_50_ lower than 50 µM. Caffeic acid phenethylamide (CAPA, compound **1**) was the most active of this series, with an IC_50_ value of 6.9 μM. Whereas the activity of nor-NOHA was retained (IC_50_ = 5.7 ± 0.6 µM) on an assay using a human recombinant arginase (h-ARG I), CAPA showed poorer activity (IC_50_ = 60.3 ± 7.8 µM) on h-ARG I compared to b-ARG-I. Therefore, although preliminary studies on b-ARG I, which is much cheaper than h-ARG I, could be useful to identify new mammalian arginase inhibitors, the most active compounds should also be tested on h-ARG I before going further with in vivo experiments. Enzyme kinetic studies, performed on b-ARG I, identified CAPA as a competitive inhibitor with K_i_ = 5.5 ± 1.0 μM. SARs and docking studies indicated the crucial role of caffeoyl moiety, which could penetrate into the active site of ARG-I in order to chelate the Mn^2+^ cofactor and interact with the important preserved residues (Asp124, His126, His141, Asp232, Asp234, and Thr246) involved in the catalytic site of arginase, thus disturbing the enzymatic activity. Overall, our results identified for the first time the important role of the caffeoyl moiety in binding the arginase active site. Considering the high IC_50_ value obtained with CAPA against h-ARG I, cinnamide derivatives could constitute potential new lead compounds for the development of arginase inhibitors with potential therapeutic applications. For instance, the phenethylamine moiety could be replaced by a part displaying specific interactions on the top of the arginase active site in order to design a new generation of cinnamides.

## Figures and Tables

**Table 1:** Structures of the synthesized compounds and their inhibitory potencies toward b-ARG I.

Compound	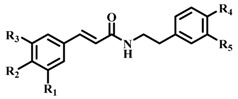					b-ARG I Inhibition ^a^ (%) (100 μM)	IC_50_^b^ (µM)
	R_1_	R_2_	R_3_	R_4_	R_5_		
**1** (CAPA)	OH	OH	H	H	H	64 ± 2	6.9 ± 1.3
**2**	OCH_3_	OH	H	H	H	37 ± 4	n.d ^c^
**3**	H	OH	H	H	H	31 ± 4	n.d
**4**	OCH_3_	OCH_3_	OCH_3_	H	H	35 ± 2	n.d
**5**	H	H	H	H	H	34 ± 2	n.d
**6**	OH	OH	H	OH	H	58 ± 2	22.1 ± 1.6
**7**	OCH_3_	OH	H	OH	H	24 ± 2	n.d
**8**	H	OH	H	OH	H	40 ± 2	n.d
**9**	OCH_3_	OCH_3_	OCH_3_	OH	H	30 ± 2	n.d
**10**	H	H	H	OH	H	34 ± 3	n.d
**11**	OH	OH	H	OH	OH	62 ± 3	114.9 ± 1.3
**12**	OCH_3_	OH	H	OH	OH	61 ± 3	198.7 ± 1.4
**13**	H	OH	H	OH	OH	54 ± 1	170.4 ± 1.7
**14**	OCH_3_	OCH_3_	OCH_3_	OH	OH	53 ± 1	193.6 ± 1.4
**15**	H	H	H	OH	OH	68 ± 3	39.3 ± 1.4
**16**	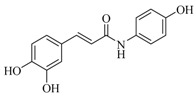					59 ± 1	35.6 ± 1.3
**17 ^d^**	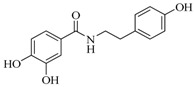					52 ± 2	175.3 ± 1.5
**18**	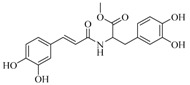					62 ± 1	41.9 ± 1.3
**19**	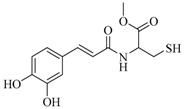					67 ± 1	37.0 ± 1.3
Caffeic acid	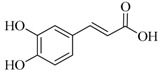					61 ± 4	86.7 ± 8.0
CGA	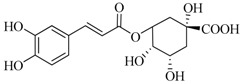					71 ± 1	10.6 ± 1.6
nor-NOHA ^e^	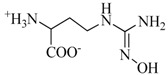					99 ± 1	1.7 ± 0.2

^a^ The values were obtained from three separate in-duplicate experiments; ^b^ IC_50_: Half maximal inhibitory concentration was obtained from three separate in-triplicate experiments; ^c^ n.d: not determined; ^d^ Compound **17** was prepared from 3,4-dihydroxybenzoic acid; ^e^ *N*^ω^-hydroxy-nor-l-arginine (nor-NOHA): used as reference inhibitor.

**Table 2:** Comparison of K_i_ values for arginase inhibition by CAPA, CGA and nor-NOHA.

Inhibitor	K_i_ (µM)	Type of Inhibition
CAPA (1)	5.5 ± 1.0	competitive
CGA	8.4 ± 1.2	competitive
nor-NOHA	1.3 ± 0.1	competitive

## References

[1] Maccallini C., Fantacuzzi M., Amoroso R. (2013). Amidine-based bioactive compounds for the regulation of arginine metabolism. Mini Rev. Med. Chem..

[2] Dimski D.S. (1994). Ammonia metabolism and the urea cycle: Function and clinical implications. J. Vet. Intern. Med. Am. Coll. Vet. Int. Med..

[3] Morris S.M. (2002). Regulation of enzymes of the urea cycle and arginine metabolism. Annu. Rev. Nutr..

[4] Caldwell R.B., Toque H.A., Narayanan S.P., Caldwell R.W. (2015). Arginase: An old enzyme with new tricks. Trends Pharmacol. Sci..

[5] Jenkinson C.P., Grody W.W., Cederbaum S.D. (1996). Comparative properties of arginases. Comp. Biochem. Physiol. B Biochem. Mol. Biol..

[6] Bagnost T., Ma L., da Silva R.F., Rezakhaniha R., Houdayer C., Stergiopulos N., André C., Guillaume Y., Berthelot A., Demougeot C. (2010). Cardiovascular effects of arginase inhibition in spontaneously hypertensive rats with fully developed hypertension. Cardiovasc. Res..

[7] Segal R., Hannan J.L., Liu X., Kutlu O., Burnett A.L., Champion H.C., Kim J.H., Steppan J., Berkowitz D.E., Bivalacqua T.J. (2012). Chronic oral administration of the arginase inhibitor 2(*S*)-amino-6-boronohexanoic acid (ABH) improves erectile function in aged rats. J. Androl..

[8] Grasemann H., Dhaliwal R., Ivanovska J., Kantores C., McNamara P.J., Scott J.A., Belik J., Jankov R.P. (2015). Arginase inhibition prevents bleomycin-induced pulmonary hypertension, vascular remodeling, and collagen deposition in neonatal rat lungs. Am. J. Physiol. Lung Cell. Mol. Physiol..

[9] Olivon V.C., Fraga-Silva R.A., Segers D., Demougeot C., de Oliveira A.M., Savergnini S.S., Berthelot A., de Crom R., Krams R., Stergiopulos N. (2013). Arginase inhibition prevents the low shear stress-induced development of vulnerable atherosclerotic plaques in ApoE−/− mice. Atherosclerosis.

[10] Morris S.M., Gao T., Cooper T.K., Kepka-Lenhart D., Awad A.S. (2011). Arginase-2 mediates diabetic renal injury. Diabetes.

[11] Meurs H., Zaagsma J., Maarsingh H., van Duin M. (2010). Use of Arginase Inhibitors in the Treatment of Asthma and Allergic Rhinitis. US.

[12] Van Zandt M.C., Whitehouse D.L., Golebiowski A., Ji M.K., Zhang M., Beckett R.P., Jagdmann G.E., Ryder T.R., Sheeler R., Andreoli M. (2013). Discovery of (*R*)-2-Amino-6-borono-2-(2-(piperidin-1-yl)ethyl)hexanoic acid and congeners as highly potent inhibitors of human arginases I and II for treatment of myocardial reperfusion injury. J. Med. Chem..

[13] Kavalukas S.L., Uzgare A.R., Bivalacqua T.J., Barbul A. (2012). Arginase inhibition promotes wound healing in mice. Surgery.

[14] Singh R., Pervin S., Karimi A., Cederbaum S., Chaudhuri G. (2000). Arginase Activity in human breast cancer cell lines: *N*^ω^-Hydroxy-l-arginine selectively inhibits cell proliferation and induces apoptosis in MDA-MB-468 cells. Cancer Res..

[15] Boucher J.L., Custot J., Vadon S., Delaforge M., Lepoivre M., Tenu J.P., Yapo A., Mansuy D. (1994). Nω-Hydroxy-l-arginine, an intermediate in the l-arginine to nitric oxide pathway, is a strong inhibitor of liver and macrophage arginase. Biochem. Biophys. Res. Commun..

[16] Custot J., Boucher J.-L., Vadon S., Guedes C., Dijols S., Delaforge M., Mansuy D. (1996). *N*^ω^-Hydroxyamino-α-amino acids as a new class of very strong inhibitors of arginases. JBIC J. Biol. Inorg. Chem..

[17] Custot J., Moali C., Brollo M., Boucher J.L., Delaforge M., Mansuy D., Tenu J.P., Zimmermann J.L. (1997). The new α-amino acid *N*^ω^-hydroxy-nor-l-arginine:  A high-affinity inhibitor of arginase well adapted to bind to its manganese cluster. J. Am. Chem. Soc..

[18] Kim N.N., Cox J.D., Baggio R.F., Emig F.A., Mistry S.K., Harper S.L., Speicher D.W., Morris S.M., Ash D.E., Traish A. (2001). Probing erectile function:  *S*-(2-Boronoethyl)-l-cysteine binds to arginase as a transition state analogue and enhances smooth muscle relaxation in human penile corpus cavernosum. Biochemistry.

[19] Baggio R., Emig F.A., Christianson D.W., Ash D.E., Chakder S., Rattan S. (1999). Biochemical and functional profile of a newly developed potent and isozyme-selective arginase inhibitor. J. Pharmacol. Exp. Ther..

[20] Ivanenkov Y.A., Chufarova N. V. (2013). Small-molecule arginase inhibitors. Pharm. Pat. Anal..

[21] Havlinova Z., Babicova A., Hroch M., Chladek J. (2013). Comparative pharmacokinetics of *N*^ω^-hydroxy-nor-l-arginine, an arginase inhibitor, after single-dose intravenous, intraperitoneal and intratracheal administration to brown Norway rats. Xenobiotica.

[22] Morris S.M. (2009). Recent advances in arginine metabolism: Roles and regulation of the arginases. Br. J. Pharmacol..

[23] Schade D., Kotthaus J., Klein N., Kotthaus J., Clement B. (2011). Prodrug design for the potent cardiovascular agent *N*^ω^-hydroxy-l-arginine (NOHA): Synthetic approaches and physicochemical characterization. Org. Biomol. Chem..

[24] Ilies M., di Costanzo L., Dowling D.P., Thorn K.J., Christianson D.W. (2011). Binding of α,α-disubstituted amino acids to arginase suggests new avenues for inhibitor design. J. Med. Chem..

[25] Golebiowski A., Beckett R.P., van Zandt M., Ji M.K., Whitehouse D., Ryder T.R., Jagdmann E., Andreoli M., Mazur A., Padmanilayam M. (2013). 2-Substituted-2-amino-6-boronohexanoic acids as arginase inhibitors. Bioorg. Med. Chem. Lett..

[26] Golebiowski A., Whitehouse D., Beckett R.P., van Zandt M., Ji M.K., Ryder T.R., Jagdmann E., Andreoli M., Lee Y., Sheeler R. (2013). Synthesis of quaternary α-amino acid-based arginase inhibitors via the Ugi reaction. Bioorg. Med. Chem. Lett..

[27] Cama E., Shin H., Christianson D.W. (2003). Design of amino acid sulfonamides as transition-state analogue inhibitors of arginase. J. Am. Chem. Soc..

[28] Ilies M., di Costanzo L., North M.L., Scott J.A., Christianson D.W. (2010). 2-Aminoimidazole amino acids as inhibitors of the binuclear manganese metalloenzyme human arginase I. J. Med. Chem..

[29] Zakharian T.Y., di Costanzo L., Christianson D.W. (2008). *S*-2-Amino-6-nitrohexanoic acid binds to human arginase I through multiple nitro−metal coordination interactions in the binuclear manganese cluster. J. Am. Chem. Soc..

[30] Shin H., Cama E., Christianson D.W. (2004). Design of amino acid aldehydes as transition-state analogue inhibitors of arginase. J. Am. Chem. Soc..

[31] Zakharian T.Y., di Costanzo L., Christianson D.W. (2008). Synthesis of (2*S*)-2-amino-7,8-epoxyoctanoic acid and structure of its metal-bridging complex with human arginase I. Org. Biomol. Chem..

[32] Girard-Thernier C., Pham T.-N., Demougeot C. (2015). The promise of plant-derived substances as inhibitors of arginase. Mini Rev. Med. Chem..

[33] Pham T., Guglielmetti A., Fimbel S., Demougeot C., Girard-Thernier C. (2014). Arginase inhibitory activity of several natural polyphenols using a novel in vitro test on purified bovine arginase. Planta Med..

[34] Bordage S., Pham T.-N., Zedet A., Gugglielmetti A.-S., Nappey M., Demougeot C., Girard-Thernier C. (2016). Investigation of mammal arginase inhibitory properties of natural ubiquitous polyphenols by using an optimized colorimetric microplate assay. Planta Med..

[35] Wang L.-N., Wang W., Hattori M., Daneshtalab M., Ma C.-M. (2016). Synthesis, Anti-HCV, antioxidant and reduction of intracellular reactive oxygen species generation of a chlorogenic acid analogue with an amide bond replacing the ester bond. Molecules.

[36] Le Mellay-Hamon V., Criton M. (2009). Phenylethylamide and phenylmethylamide derivatives as new tyrosinase inhibitors. Biol. Pharm. Bull..

[37] Okombi S., Rival D., Boumendjel A., Mariotte A.-M., Perrier E. (2007). Para-Coumaric Acid or Para-Hydroxycinnamic Acid Derivatives and Their Use in Cosmetic or Dermatological Compositions. US.

[38] Coste J., Le-Nguyen D., Castro B. (1990). PyBOP: A new peptide coupling reagent devoid of toxic by-product. Tetrahedron Lett..

[39] Berndt M., Hölemann A., Niermann A., Bentz C., Zimmer R., Reissig H.-U. (2012). Replacement of HMPA in Samarium Diiodide Promoted Cyclizations and Reactions of Organolithium Compounds. Eur. J. Org. Chem..

[40] Corraliza I.M., Campo M.L., Soler G., Modolell M. (1994). Determination of arginase activity in macrophages: A micromethod. J. Immunol. Methods.

[41] Cornish-Bowden A. (1974). A simple graphical method for determining the inhibition constants of mixed, uncompetitive and non-competitive inhibitors (Short Communication). Biochem. J..

[42] Yung-Chi C., Prusoff W.H. (1973). Relationship between the inhibition constant (KI) and the concentration of inhibitor which causes 50 per cent inhibition (I50) of an enzymatic reaction. Biochem. Pharmacol..

[43] Di Costanzo L., Pique M.E., Christianson D.W. (2007). Crystal structure of human arginase I complexed with thiosemicarbazide reveals an unusual thiocarbonyl μ-sulfide ligand in the binuclear manganese cluster. J. Am. Chem. Soc..

[44] BioSolveIT–GmbH (2012). FlexX (Protein–Ligand Docker) user & technical reference as part of LeadIT 2.0. https://www.biosolveit.de/FlexX.

[45] Weng Y.-C., Chuang C.-F., Chuang S.-T., Chiu H.-L., Kuo Y.-H., Su M.-J. (2012). KS370G, a synthetic caffeamide derivative, improves left ventricular hypertrophy and function in pressure-overload mice heart. Eur. J. Pharmacol..

[46] Shi Z.-H., Li N.-G., Shi Q.-P., Hao-Tang B.S.P., Tang Y.-P., Wei-Li B.S.P., Lian-Yin B.S.P., Yang J.-P., Duan J.-A. (2013). Design, synthesis and biological evaluation of ferulic acid amides as selective matrix metalloproteinase inhibitors. Med. Chem..

[47] Yamazaki Y., Kawano Y., Uebayasi M. (2008). Induction of adiponectin by natural and synthetic phenolamides in mouse and human preadipocytes and its enhancement by docosahexaenoic acid. Life Sci..

[48] Yao H., Tang Y., Yamamoto K. (2012). Metal-free oxidative amide formation with *N*-hydroxysuccinimide and hypervalent iodine reagents. Tetrahedron Lett..

[49] Hong S.S., Jeong W., Kwon J.G., Choi Y.-H., Ahn E.-K., Ko H.-J., Seo D.-W., Oh J.S. (2013). Phenolic Amides from the Fruits of Tribulus terrestris and Their Inhibitory Effects on the Production of Nitric Oxide. Bull. Korean Chem. Soc..

[50] Kan S., Chen G., Han C., Chen Z., Song X., Ren M., Jiang H. (2011). Chemical constituents from the roots of *Xanthium sibiricum*. Nat. Prod. Res..

[51] Lin C.-F., Lay H.-L., Ni C.-L., Chen C.-C. (2013). Phenolic Components of *Dendrobium antennatum*. Chem. Nat. Compd..

[52] Tamiz A.P., Cai S.X., Zhou Z.-L., Yuen P.-W., Schelkun R.M., Whittemore E.R., Weber E., Woodward R.M., Keana J.F.W. (1999). Structure−Activity relationship of *N*-(Phenylalkyl)cinnamides as novel NR2B subtype-selective NMDA receptor antagonists. J. Med. Chem..

[53] Son S., Lewis B.A. (2001). Free radical scavenging and antioxidative activity of caffeic acid amide and ester analogues:  Structure−activity relationship. J. Agric. Food Chem..

[54] Wu Z., Zheng L., Li Y., Su F., Yue X., Tang W., Ma X., Nie J., Li H. (2012). Synthesis and structure–activity relationships and effects of phenylpropanoid amides of octopamine and dopamine on tyrosinase inhibition and antioxidation. Food Chem..

[55] Michalet S., Cartier G., David B., Mariotte A.-M., Dijoux-franca M.-G., Kaatz G. W., Stavri M., Gibbons S. (2007). *N*-Caffeoylphenalkylamide derivatives as bacterial efflux pump inhibitors. Bioorg. Med. Chem. Lett..

[56] Shi Z.-H., Li N.-G., Shi Q.-P., Tang H., Tang Y.-P., Li W., Yin L., Yang J.-P., Duan J.-A. (2012). Design, synthesis and biological evaluation of caffeic acid amides as selective MMP-2 and MMP-9 inhibitors. Drug Dev. Res..

[57] Chou S.-C., Su C.-R., Ku Y.-C., Wu T.-S. (2009). The constituents and their bioactivities of *Houttuynia cordata*. Chem. Pharm. Bull..

[58] Larsson R., Blanco N., Johansson M., Sterner O. (2012). Synthesis of C-1 indol-3-yl substituted tetrahydroisoquinoline derivatives via a Pictet–Spengler approach. Tetrahedron Lett..

[59] Lee S.U., Shin C.-G., Lee C.-K., Lee Y.S. (2007). Caffeoylglycolic and caffeoylamino acid derivatives, halfmers of l-chicoric acid, as new HIV-1 integrase inhibitors. Eur. J. Med. Chem..

[60] SYBYL-X, Tripos International. https://www.certara.com.

[61] Rarey M., Kramer B., Lengauer T., Klebe G. (1996). A fast flexible docking method using an incremental construction algorithm. J. Mol. Biol..

[62] Chemical Computing Group Inc. Molecular Operating Environment (MOE). https://www.chemcomp.com.

